# Preparation and Characterization of Sn Micro- and Nanoparticles

**DOI:** 10.3390/nano16130825

**Published:** 2026-07-05

**Authors:** Alena Michalcová, Šárka Msallamová, Dominika Fink, Olga Hrubá, Anna Boukalová, Tomáš Balický, Jan Rohlíček

**Affiliations:** 1 Department of Metals and Corrosion Engineering, University of Chemistry and Technology in Prague, Technická 5, 166 28 Prague 6, Czech Republic; msallams@vscht.cz (Š.M.); hrubap@vscht.cz (O.H.); boukaloa@vscht.cz (A.B.); balickyt@vscht.cz (T.B.); 2 Division of Solid State Physics, Institute of Physics of the Czech Academy of Sciences, Cukrovarnická 10/112, 162 00 Prague 6, Czech Republic; rohlicek@fzu.cz

**Keywords:** tin, phase transformation, tin pest, precipitation

## Abstract

This study investigates the preparation and characterization of tin micro- and nanoparticles with an emphasis on phase-transformation-induced particle formation and chemical purity. Microparticles were generated through repeated phase transformations between β-Sn (white tin) and α-Sn (gray tin), exploiting the associated volumetric changes to induce fragmentation and particle size reduction. The evolution of particle size distribution was systematically analyzed as a function of transformation cycles. The data were analyzed using the modified Johnson–Mehl–Avrami–Kolmogorov equation, and the saturation particle size corresponds to the grain size of the original tin sheet. The phase transformation was induced homogeneously by α-Sn particles and heterogeneously by InSb, and the results were comparable. The influence of the surrounding atmosphere was studied. The increase in oxygen content during repeated phase transformation was measured. In parallel, tin nanoparticles were synthesized via a solution-based route using ammonium hexachlorostannate as a precursor. The nanoparticles precipitated from this solution at mild temperatures during the β-Sn to α-Sn transformation at 13.2 °C. Both micro- and nanoparticles were characterized in terms of morphology and size distribution. The results provide insight into the relationship between phase transformation and particle size reduction mechanisms, and offer a controllable pathway for the preparation of tin particles across micro- and nanoscale regimes.

## 1. Introduction

Tin (Sn) is a fundamental post-transition metal with a historical prominence in metallurgical frameworks, most notably as a crucial constituent in lead-free solder alloys and electronic packaging. Characterized by its relatively low melting point in bulk form (232 °C) and environmental friendliness compared to heavy metals, tin has attracted significant modern research interest due to its crystal structure-dependent properties [1,2]. At standard ambient conditions, tin primarily exists as white tin (β-Sn), a metallic phase exhibiting a body-centered tetragonal structure that is thermodynamically stable down to 13.2 °C [1,2]. Upon cooling below this critical temperature threshold, it undergoes an allotropic phase transformation into gray tin (α-Sn), which exhibits a diamond cubic crystal structure [2]. This phase transformation is also known as tin pest. The mechanism of this phase transformation is most probably massive in nature, although it never takes place completely [3]. While bulk alpha-Sn behaves as a zero-gap semimetal protected by the cubic symmetry of its lattice, the application of external spatial perturbations, such as strain engineering or nanoscale quantum confinement, can effectively modify its nontrivial electronic structure, transforming it into a three-dimensional topological Dirac semimetal (TDS) [2,4].

Owing to these unique phase-dependent physicochemical characteristics, both alpha- and beta-phase micro- and nanoparticles find widespread utility across diverse technological and biomedical frontiers. Tetragonal beta-Sn particles are highly valued in technological systems, serving as efficient liquid metal binders to lower sintering temperatures in flexible conductive printing inks [1,5], as field-emission enhancers when combined with graphene frameworks [6], and as high-capacity anode materials for next-generation lithium-ion and sodium-ion energy storage systems [2,6]. Concurrently, tin formulations have been emerging in biomedical applications, notably as anti-corrosive, hydrogen-suppressing agents in biodegradable temporary implants, and in the form of organotin complexes displaying strong antitumor activity during in vitro and in vivo profiling [1,2,7]. More recently, free-standing alpha-Sn nanocrystals have revolutionized the field of nanomedicine and cellular theranostics [2]. When restricted below the exciton Bohr radius (~12.59 nm), alpha-Sn undergoes a quantum-size-induced bandgap opening [2], enabling exceptional near-infrared (NIR) light absorption and a high photothermal conversion efficiency (~42.4%) that makes it an optimal agent for non-invasive, renal-clearable photothermal cancer therapies [2].

The practical implementation of these nanomaterials necessitates precise control over their synthesis pathways to dictate particle morphology, size distribution, and crystalline phase configuration. For beta-Sn and general tin oxide nanoparticles, synthetic methodologies predominantly rely on a modified polyol process—utilizing precursors such as tin(II) 2-ethylhexanoate or tin(II) acetate coupled with a strong reducing agent (NaBH_4_) in a glycol medium—which allows for temperature-dependent fine-tuning of particle diameters [1,5]. Alternative fabrication techniques include solution plasma synthesis from metallic wire [6], wet chemical reduction assisted by aluminum powder [7], and direct thermal evaporation onto flexible phospholipid membranes to generate large-scale self-assembled monolayers [8]. On the other hand, the synthesis of gray alpha-Sn has traditionally been restricted to high-cost physical methods, such as molecular beam epitaxy (MBE) to grow thin single-crystal epilayers on lattice-matched substrates (InSb or CdTe) via compressive strain engineering [2,4].

However, recent breakthroughs have established more sustainable, non-conventional routes, including microplasma fabrication [1] and room-temperature aqueous phase wet reduction using a PEG 4000 capping polymer [2]. This latter approach effectively limits surface energy and arrests the alpha -> beta backward transformation, successfully yielding free-standing, highly biocompatible alpha-Sn nanostructures without requiring complex substrate assistance [2].

The preparation of high-purity Sn particles by repeated β-Sn to α-Sn phase transformation was already described by [9]. In our work, we have quantified the amount of trapped gases in the powder materials in detail and also analyzed the dependence of particle size on the modified Johnson–Mehl–Avrami–Kolmogorov equation traditionally used for the description of grain size refinement by severe plastic deformation [10,11,12]. The β-Sn to α-Sn phase transformation has already been described to follow the Avrami equation [13].

Ammonium hexachlorostannate—(NH_4_)_2_SnCl_6_—is also known as a pink salt although it is of white color. The origin of the name is in its usage as a mordant for pink and scarlet coloring of textiles [14]. It used to be used to accelerate and unify the β-Sn to α-Sn phase transformation [15,16]. The mechanism of its function was widely studied [17,18] but never fully understood.

## 2. Materials and Methods

The 5N Sn (Goodfellow, Huntingdon, UK) was used in all experiments. To observe the initial grain size, the sheet was polished by a Leica RES 102 ion polisher (Leica Mikrosysteme, Wetzlar, Germany), and the sample was observed by SEM TESCAN VEGA 3 LMU (TESCAN, Brno, Czech Republic) equipped by EDS and BESD analyzers operated by Aztec by Oxford Instruments (Oxford Instruments, High Wycombe, UK). The TEM observations were performed by Jeol 2200 FS (Tokyo, Japan) working at 200 kV, equipped with an EDS Aztec analyzer by Oxford Instruments (Oxford Instruments, High Wycombe, UK) and a TVIPS camera (TVIPS, Gauting, Germany). The image analysis was performed by ImageJ software (1.37 version, Bethesda, MD, USA). Due to the irregular shape of particles and in some cases their binodal distribution of sizes, the analyses were performed manually: two perpendicular lengths were measured for each particle and their average was estimated as the particle size.

The XRD was measured by a powder diffractometer Empyrean PANalytical B.V., Almelo, The Netherlands. The goniometer Omega/2 Theta was used for the measurement in the range 4–50° with a step of 0.013° and dwell time of 60 s. The Cu anode was used with a current of 40 mA. A nickel filter with a thickness of 20 µm was used during the measurement. Raman microscopy/spectroscopy was measured by a Thermo Scientific DXR Raman Microscope (Thermo Fisher Scientific, Waltham, MA, USA). The contents of oxygen and nitrogen in samples were analyzed by a Galileo G8 Gas Analyzer (Bruker Elemental GmbH, Kalkar, Germany). The oxygen content was analyzed on an equivalent machine in the laboratory of Czech Technical University to prove the reliability of results. The chemical compositions of ethanol-based solutions were analyzed by liquid chromatography and mass spectroscopy using Orbitrap Exploris 240 (Thermo Fisher Scientific, Waltham, MA, USA, Orbitrap Exploris 240). The Sn^2+^ presence was detected by coupling ICP-MS with liquid chromatography (LC–ICP-MS) performed by PerkinElmer NexION 350D (PerkinElmer, Woodbridge, ON, Canada).

The phase transformation was induced by storing the inoculated samples at −50 °C in a TEFCOLD freezer (TEFCOLD, Vítkovice, Czech Republic). The temperature of −50 °C was chosen as the condition of the highest phase transformation rate [19,20]. The inoculation with α-Sn or InSb was done with a load of 100 N.

The samples with pink salt were stored in a refrigerator at temperatures of 7 °C and −18 °C for the freezer. The pink salt was supplied in purity of 98% by Fisher Scienticif (Pardubice, Czech Republic). The solubility of pink salt in ethanol is low [14], but it was documented that a 0.001% solution is still affecting the phase transformation [18]. In this study, 0.5 g in 200 mL of ethanol in the form of an oversaturated solution was used (the solution above residual crystals was used to ensure saturation independently of temperature).

Experimental data were fitted in Origin 2019 to obtain the coefficients for the modified Johnson–Mehl–Avrami–Kolmogorov equation.

## 3. Results and Discussion

**Figure 1:**
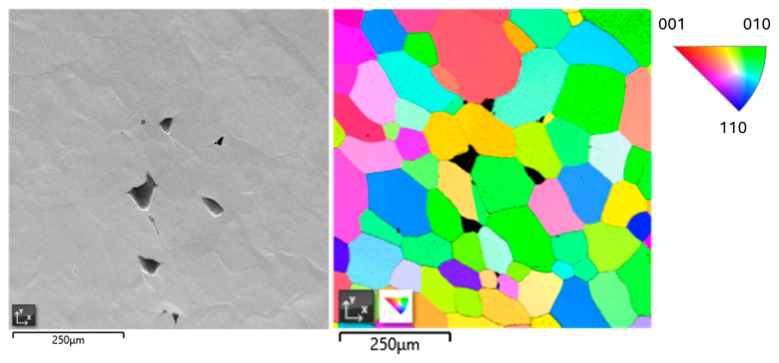
EBSD map of initial Sn sheet.

This 5N tin sheet was characterized before the beginning of further experiments. To determine the size, shape and orientation of grains, EBSD analysis was performed. Figure 1 shows an IPF map of the Sn sheet after the ion polishing process. The grains are almost equiaxed with slight residual signs of the rolling process. The grains are randomly oriented. The grain size (evaluated as equivalent circle diameter) reaches a value of 79 ± 43 µm. The black (unindexed) parts are caused by the polishing process—in these places the Sn sheet was thinned to disappearance.

A TEM sample was prepared from the Sn sheet. Subsequently, the sample was inoculated with InSb and stored at −50 °C for 24 h. These conditions ensure the occurrence of phase transformation not only in the inoculated place but also in the vicinity [21].

**Figure 2:**
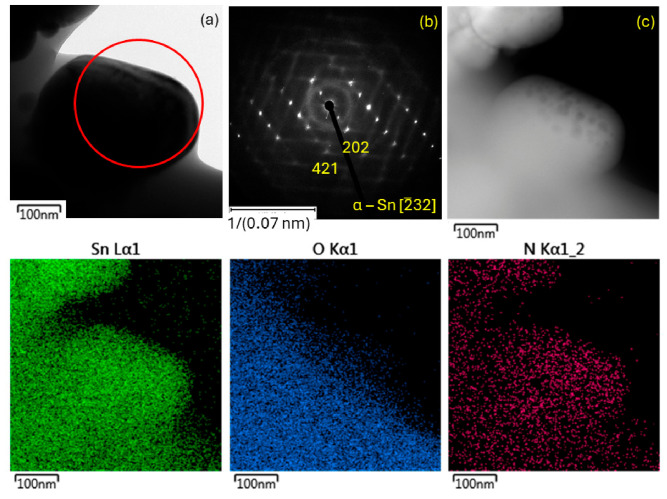
TEM micrograph of Sn sheet after 24 h at −50 °C; (**a**) TEM-BF; (**b**) SAED; (**c**) STEM-DF and elemental maps. α-Sn particle labelled by red circle.

Figure 2 shows the formation of a roundish particle in the sample. This particle was identified by SAED from the area labeled by the red circle as α-Sn. The material contrast shown in STEM-DF (Figure 2c) is negligible. Surprisingly, in the α-Sn particle, an increased content of oxygen was not detected. On the other hand, the content of nitrogen seems to be higher predominantly in the α-Sn. This is in disagreement with [21], where the stabilization of α-Sn by the presence of oxygen is described.

**Figure 3:**
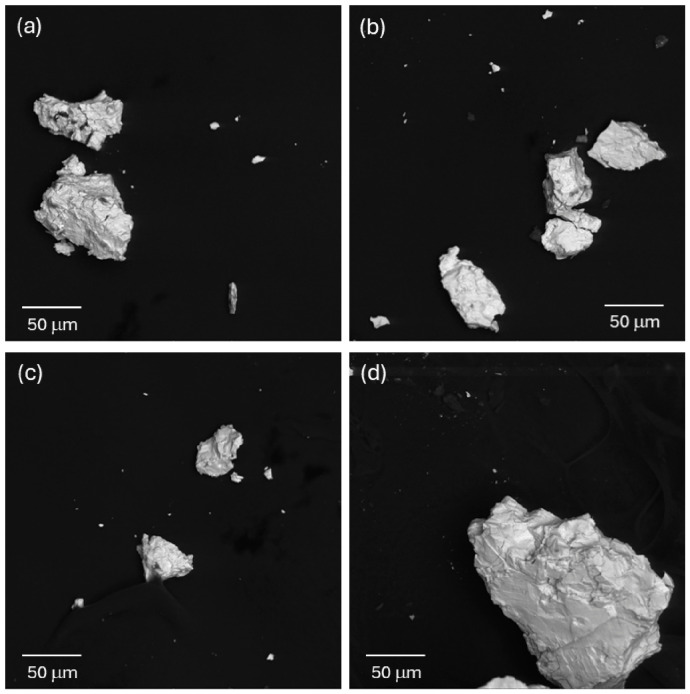
SEM micrographs of Sn after phase transformation in (**a**) air; (**b**) nitrogen; (**c**) Ar and (**d**) evacuated ampoule.

**Figure 4:**
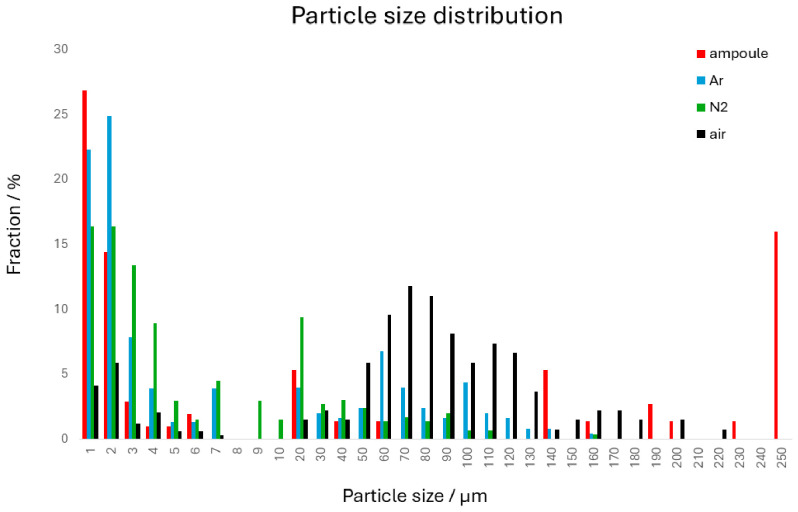
Particle size distribution after transformation in different atmospheres.

Phase transformation of Sn sheet after inoculation by InSb was performed in four different atmospheres—in air (the sample was placed freely in a zipper plastic bag), in nitrogen and argon (in both cases the sample was placed in two layers of zipper plastic bag, each filled with protective gas), and in an evacuated ampoule made from silicon glass. The differences in size of formed particles are clearly visible in Figure 3. To have statistical information, the particle sizes were measured, and the histograms are given in Figure 4. As almost all of the samples contained a binodal distribution of particles, the *x*-axis of the plot is composed of two parts, 1 to 10 µm and 10 to 250 (and more) µm. In [9], a continuous decrease in particle size down to 5 µm was observed with lower oxygen contamination by alternating air and vacuum. Our results show that this approach can also influence the particle size.

The content of contamination gases in Sn particles was measured, and the results are given in Table 1.

The values given in Table 1 exhibit significant fluctuation, independent of the conditions of sample preparation or of the machine used for measuring. The explanation might be that the β-Sn to α-Sn phase transformation is not a homogeneous process and the amount taken for analysis (approx. 100 mg) is too low to average the fluctuations. Nevertheless, some trends are clearly visible:

- The amount of oxygen increases with the number of transformations in the air;
- The amount of oxygen is significantly higher in non-air atmospheres;
- The amount of nitrogen increases only by transformation in a nitrogen atmosphere.

The last-mentioned point is probably caused by adsorption of nitrogen on the surface of particles. The first point is in agreement with previously observed increases of oxygen content with the number of cycles of the β-Sn to α-Sn phase transformation [9]. The increase of oxygen content was also observed in [9]. In our case, it might be caused by the presence of residual SiO_2_ from the ampoule for measurements 13 and 14 in Table 1. Measurement 12 was performed on a smaller amount of precisely separated Sn and still exhibits high oxygen content but no nitrogen content. The explanation might be the formation of a self-protecting SnO_2_ layer in the air, while in other atmospheres the Sn surface is naked and serves as a sponge for oxygen after the opening of the vessel with a protective atmosphere. Based on this fact and also the most normal distribution of particle size (as shown in Figure 4), all following experiments were performed in the air.

**Figure 5:**
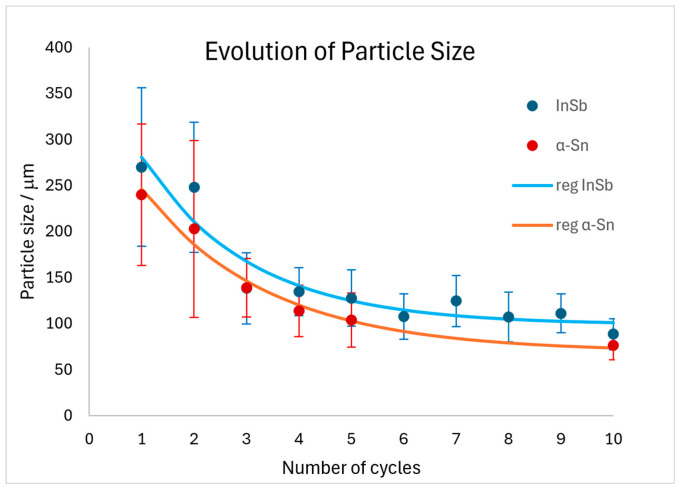
Dependence of particle size on number of β-Sn to α-Sn phase transformation cycles.

Two series of samples were studied for the particle size dependence on the number of β-Sn to α-Sn phase transformation cycles—one inoculated by α-Sn and the other one by InSb—and the results (marks) and corresponding fitted curves (lines labeled as “reg”) are given in the plot in Figure 5. In both cases, the particle size is rapidly decreasing for the first three or four cycles and further stabilizes. The data can be fitted by size using the modified Johnson–Mehl–Avrami–Kolmogorov equation traditionally used for the description of grain size refinement by severe plastic deformation [10,11,12]. The β-Sn to α-Sn phase transformation itself (the percentage of transformed fraction of the sample) is usually described by the Avrami equation [13,22,23] or by the modified Johnson–Mehl–Avrami–Kolmogorov equation [10] that might lead to confusion. In our case, we have used the equation in the form given in (1):

d = d_sat_ + (d_0_ − d_sat_)·e^−kn^ (1)

where d is the actual particle size, d_0_ is the initial particle size, d_sat_ is the saturated particle size, k is the material and geometry constant, and n is the number of cycles. As the initial particle size is not known and could be dependent on the size of the sample, we left it as a free parameter for fitting as well, and the results are given in Table 2.

The results are in good agreement within experimental errors. If we still want to explain the differences between α-Sn and InSb inoculated samples, we can consider the decrease in particle size of the inoculator happening for α-Sn [21], as well as the fact that the initial α-Sn particles cannot be distinguished from freshly forming particles. On the other hand, the particle size of InSb remains the same and could be easily separated by SEM-BSE observations. In both cases, the saturated particle size is far beyond the nanoscale.

**Figure 6:**
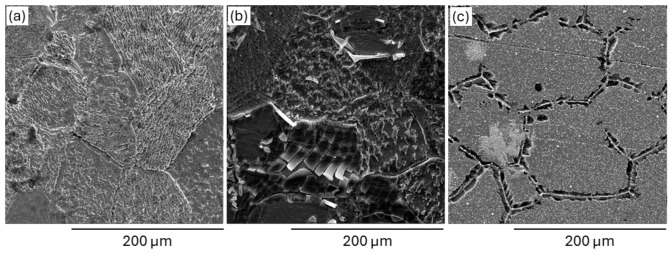
SEM-SE micrographs for 5N Sn sheet after 24 h immersion in (**a**) aqueous solution of (NH_4_)_2_SnCl_6_ at 7 °C; (**b**) ethanol solution of (NH_4_)_2_SnCl_6_ at room temperature; (**c**) ethanol solution of (NH_4_)_2_SnCl_6_ at 7 °C.

In the historical literature, the positive influence of an ethanol solution of (NH_4_)_2_SnCl_6_ on the β-Sn to α-Sn phase transformation has been described [15,16,17,18]. We have tested the immersion of a 5N Sn sheet into an aqueous solution of (NH_4_)_2_SnCl_6_ at 7 °C and an ethanol solution of (NH_4_)_2_SnCl_6_ at room temperature, 7, and −18 °C. The results for temperatures 7 and −18 °C were comparable, so only the one exposed at 7 °C is shown in Figure 6.

**Figure 7:**
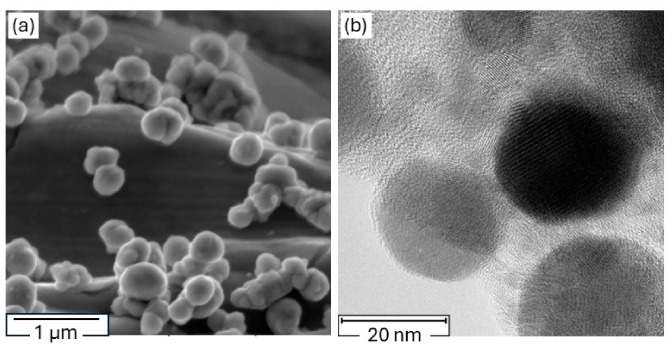
Detailed Sn particle micrograph (**a**) SEM; (**b**) TEM created by precipitation of Sn in ethanol solution of (NH_4_)_2_SnCl_6_ at 7 °C.

Figure 6 documents that the immersion in aqueous solution led only to etching of the Sn surface. The immersion in ethanol solution at room temperature (above transformation temperature) led to etching and creation of dark surface oxide layers. Immersion in ethanol solution at 7 °C (below transformation temperature) led to grain boundary etching and precipitation of small particles on the surface. The detailed view of these particles is given in Figure 7a by SEM on the Sn surface and Figure 7b by TEM after wiping the particles off the surface.

**Figure 8:**
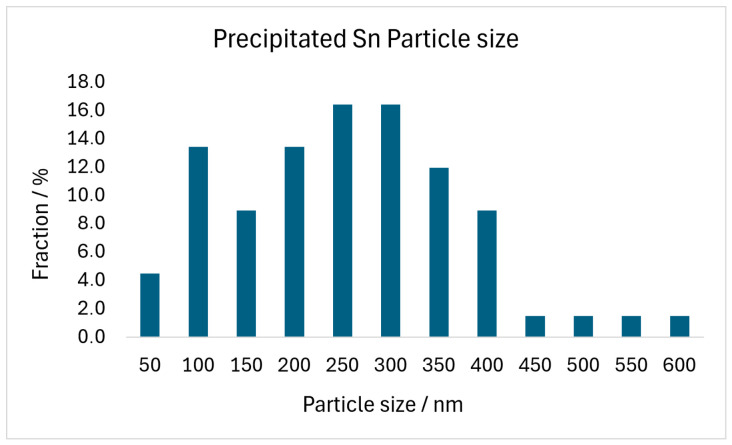
Particle size distribution of precipitated Sn particles in ethanol solution of (NH_4_)_2_SnCl_6_ at 7 °C.

The particles were approximately 100–400 nm in size, as shown in the particle size distribution plot in Figure 8.

**Figure 9:**
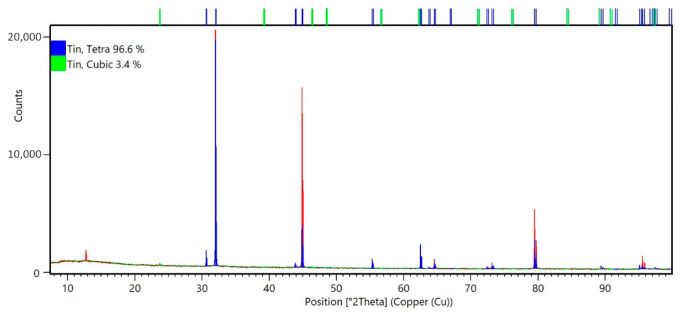
XRD of the 5N Sn sheet after 24 h immersion in ethanol solution of (NH_4_)_2_SnCl_6_ at 7 °C.

To prove the composition of particles, XRD was performed and the result is given in Figure 9. The particles form 3.4% of α-Sn on the β-Sn sheet.

**Figure 10:**
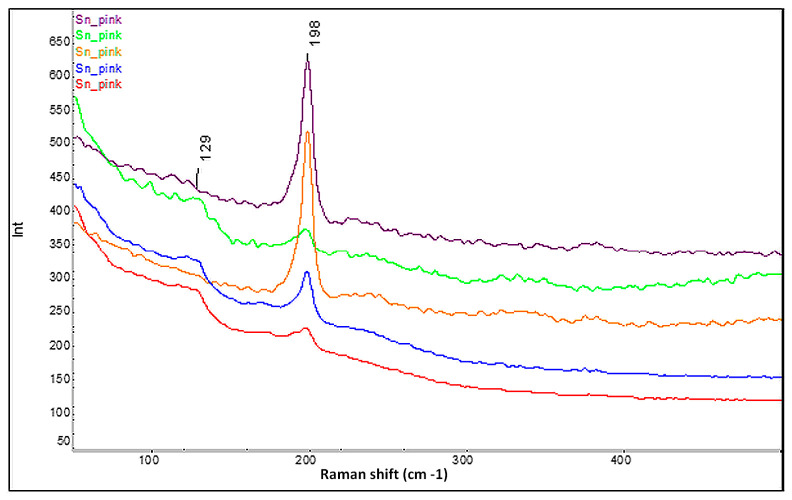
Raman spectra for several Sn particles precipitated on the 5N Sn sheet after 24 h immersion in ethanol solution of (NH_4_)_2_SnCl_6_ at 7 °C.

To obtain more localized information, the individual dark Sn particles were analyzed by Raman microscopy and the results are shown in Figure 10. In all cases, the particles were formed by α-Sn.

**Figure 11:**
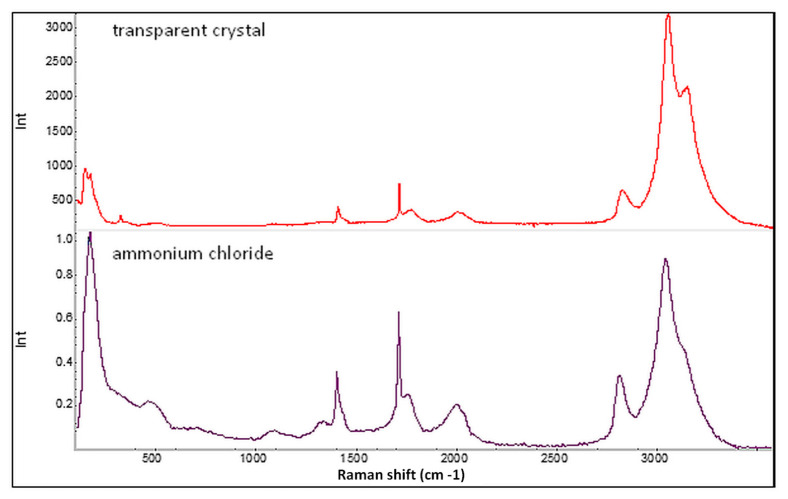
Raman spectra of transparent crystal.

Besides the α-Sn particle, the evolution of gas (increase in volume inside the zipper bag, where the reaction took place) was observed along with the formation of transparent crystals in the reaction mixture. These crystals were analyzed by Raman microscopy, and it was proven that they are composed of NH_4_Cl ammonium chloride, as shown in Figure 11.

**Figure 12:**
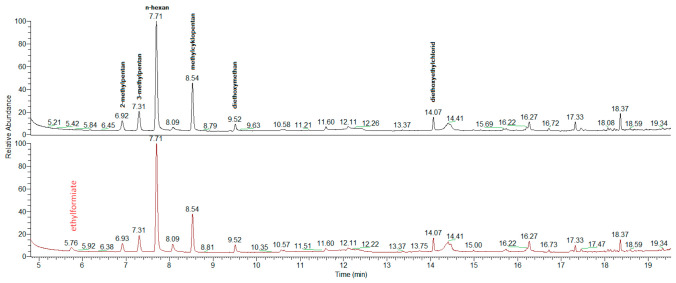
Mass spectroscopy of ethanol solution of (NH_4_)_2_SnCl_6_ before and after reaction at 7 °C.

The liquid phase of the reaction mixture was analyzed by mass spectroscopy. The difference in composition before reaction with the Sn sheet and after is not significant, but the presence of ethylformiate after the reaction was detected, as shown in Figure 12.

**Figure 13:**
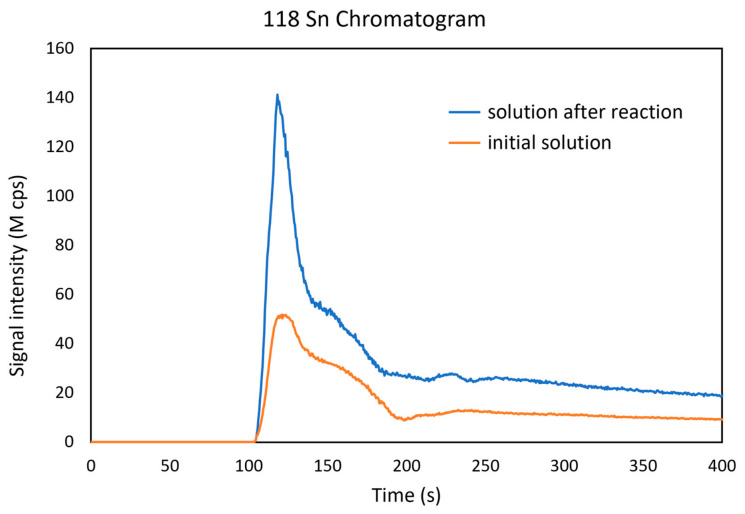
Chromatogram of 118 Sn (ICP-MS) ethanol solution of (NH_4_)_2_SnCl_6_ before and after reaction at 7 °C.

The solutions were further analyzed by ICP-MS to prove the presence of Sn^2+^ ions formed by dissolution of a 5N Sn sheet in the reaction mixture. The chromatogram shown in Figure 13 shows that no Sn^2+^ ions were detected, as they would have shorter retention times [24].

Based on all the above-mentioned results, we suggest that the interaction of the ethanol solution of (NH_4_)_2_SnCl_6_ is not the catalysis of β-Sn to α-Sn phase transformation, but it is a chemical reaction described by Equation (2)

2 CH_3_CH_2_OH + 5 Sn^4+^ + 6 H_2_O → 2 HCOOH + 2 CO_2_ + 5 Sn^0^ + 20 H^+^ (2)

This reaction might also be facilitated by the catalytic effect of Sn [25] or SnO_2_ [26] that was already observed. A small amount of α-Sn precipitate on the β-Sn surface appears dark and can be mistaken for the phase transformation. In a closer look, the surface morphology is different, etched, and missing typical blisters [21]. This precipitation process is promising for preparation of α-Sn without preferential orientation, which is common by epitaxial growth—currently the most common α-Sn preparation method [27,28].

## 4. Conclusions

The repeated β-Sn to α-Sn phase transformation leads to a decrease in particle size that can be described by the modified Johnson–Mehl–Avrami–Kolmogorov equation. The parameters of this equation are independent of the used inoculator—α-Sn or InSb. The saturated particle size is in dozens of micrometers.

The alternative route of Sn nanoparticles preparation was found to be exposure of a Sn sheet to an ethanol solution of (NH_4_)_2_SnCl_6_ under the α-Sn to β-Sn transformation temperature of 13.2 °C. Nanoparticles with a size of 100–400 nm were precipitated on the β-Sn sheet surface. The precipitation is accompanied by grain boundary etching and chemical reaction.

The function of (NH_4_)_2_SnCl_6_ in the tin phase was explained—it does not serve as a catalyst, but its presence completely changes the behavior of Sn material.

## Figures and Tables

**Table 1:** Oxygen and nitrogen content in Sn samples (Melt-extraction Analyzer).

Experiment Number	Number of Transformations	Atmosphere	Oxygen [ppm]	Nitrogen [ppm]	Laboratory
1	0		132	n.a.	CTU
2	0		184	n.a.	CTU
3	0		182	n.a.	CTU
4	0		195	n.a.	UCT
5	0		95	n.a.	UCT
6	0		159	329	UCT
7	1	Air	282	n.a.	CTU
8	1	Air	136	n.a.	CTU
9	1	Air	173	n.a.	CTU
10	1	Air	286	n.a.	UCT
11	1	Air	239	17	UCT
12	1	Vacuum	7065	n.d.	UCT
13	1	Vacuum	251,485	250	UCT
14	1	Vacuum	12,458	41	UCT
15	1	N_2_	2693	3721	UCT
16	1	N_2_	2330	4444	UCT
17	1	Ar	1896	n.d.	UCT
18	10	Air	906	n.a.	CTU
19	10	Air	1481	n.a.	CTU
20	10	Air	1261	n.a.	CTU
21	10	Air	1298	n.a.	UCT

n.a. means not analyzed, n.d. means not detected.

**Table 2:** Fitted parameters for the modified Johnson–Mehl–Avrami–Kolmogorov Equation (1).

Inoculation	d_0_ [µm]	d_sat_ [µm]	k	R^2^
α-Sn	337.05 ± 26.87	69.63 ± 13.99	0.415 ± 0.09	0.97877
InSb	394.31 ± 53.21	98.92 ± 13.46	0.485 ± 0.15	0.91374

## Data Availability

The data used within this manuscript can be accessed through the Zenodo repository: https://doi.org/10.5281/zenodo.20488226.
